# Effective Personality as a Protective Factor in Teachers’ Occupational Health

**DOI:** 10.3390/ijerph19052907

**Published:** 2022-03-02

**Authors:** Cristina Di-Giusto, María Eugenia Martín-Palacio, Marta Soledad García-Rodríguez, Francisco Javier Sánchez-Sánchez, Raquel de la Fuente-Anuncibay, Andrés Fernando Avilés-Dávila, Cesáreo Gabriel García-Rodríguez

**Affiliations:** 1 Department of Educational Sciences, University of Burgos, 09001 Burgos, Spain; cdi@ubu.es (C.D.-G.); raquelfa@ubu.es (R.d.l.F.-A.); 2 Department of Research and Psychology in Education, University Complutense of Madrid, 28040 Madrid, Spain; andresav@ucm.es; 3 Departament of Education Sciences, University of Oviedo, 33005 Oviedo, Spain; martagar@uniovi.es; 4 Department of Education and Culture of the Principality of Asturias, 33007 Oviedo, Spain; franciscojavier.sanchezsanchez@asturias.org; 5 Department of Web Application Development, ESIC (Busicess School), Pozuelo de Alarcón, 28223 Madrid, Spain; cgarcia_1963@hotmail.com

**Keywords:** effective personality, occupational health, satisfaction, teacher

## Abstract

In recent decades, there has been a growing body of research showing the relationship between teaching work and several health problems, both physical and psychological. Some of these studies relate personal competencies and resources to teachers’ occupational health. Based on the construct of Effective Personality, proposed by Martin del Buey, Martín Palacio, and Di Giusto, the aim was to analyse the relationship between the dimensions of the construct and Teachers’ Occupational Health. A descriptive cross-sectional design was used. It was based on the application of the Teacher Health Questionnaire (CSD) and the Efficacy Personality Questionnaire-Adults (CPE-A). The sample consisted of 700 non-university teachers aged between 26 and 66 years, *M* = 47.65 *SD* = 8.68. Descriptive, correlational, linear regression, and structural equation analyses were carried out. The results confirmed the relationship between the Efficacy Personality construct and Teachers’ Occupational Health (*r* = 0.45 **). In addition, the regression analysis indicated the relevance of each factor of Efficacy Personality in the factors of Teachers’ Occupational Health. The variance of Self-efficacy is the most explained by the dimensions of Efficacy Personality (40.2%), with positive relationships. The structural equation analysis confirmed the influence between Efficacy Personality and the factors of Self-Efficacy and Satisfaction, explaining 55.0% of the variance. It is concluded, therefore, that Efficacy Personality has a protective function on Teacher Occupational Health; the higher the Efficacy Personality scores are, the better the results in health gain—Self-efficacy and satisfaction—and the lower the result in health loss—burnout, cognitive affections, musculoskeletal affections, and voice alterations. These results facilitate the design of prevention and intervention programmes for teachers’ occupational health, which strengthen and improve personal and socio-affective competencies.

## 1. Introduction

The Healthy Work Environment model proposed by the World Health Organisation [1], proposes four levels of intervention to protect health, safety, and well-being in the workplace: the physical work environment, the psychosocial environment, personal health resources, and the participation of the workplace/company/organisation in the community. However, despite the time that has elapsed, most of the actions have been developed with strategies that focus mainly on the physical work environment and workplace participation in the community, but hardly any action is taken on the psychosocial work environment and personal health resources.

In the field of the teaching profession, studies have focused mainly on variables such as emotional intelligence [2,3,4,5,6,7,8,9].

Psychology and the social sciences have highlighted the importance of studying these variables, pointing out the importance of also analysing the role that other personal resources and variables can play in well-being and health at work. In the field of the teaching profession, greater emphasis has traditionally been placed on organisational variables; however, there is currently a demand for greater integration of work and personal variables to explain psychosocial risk factors at work and their consequences. In this sense, some authors [10] argue that most research has focused mainly on analysing aspects of the work environment, neglecting other aspects such as the internal functioning of the stress process and, to a lesser extent, personality variables.

In this sense, some authors point out the importance of developing personal competencies and resources to achieve healthy work organisations [11,12,13,14,15].

Attention to personal resources is a necessity within the framework of the current Healthy Work Environment model, which defines the work environment as being in which workers and employers collaborate, with the use of a continuous improvement process being important to protect the health, safety, and well-being of all employees and the sustainability of the workplace [1]. Moreover, this interest is supported by the current national and European Union (EU) social policy and the current legal system, namely the Law 31/1995 of 8 November 1995 on the Prevention of Occupational Risks [16], as well as its provisions, calling for the strengthening of personal resources as the main objective to achieve an improvement in health and well-being at work. Together, with the improvement of organisational and work-related teaching variables, it is necessary to focus on the development of personal competencies and resources that can play a moderating and controlling role in health risk factors.

Our work aimed to contribute to this demand in the teaching field. To this end, we analysed the relationship between various personal resources (Self-esteem, Self-concept, Motivation, Attribution, Expectations, Coping with Problems, Decision-making, Communication, Empathy, and Assertiveness) integrated in the Efficient Personality construct proposed by Martin Del Buey, Martinpalacio, and Di Giusto, and Occupational Health, the construct for which the model of Fernández-Puig, Longas, Chamarro, and Virgili [17] is assumed, both in its manifestations of health loss (Exhaustion, Cognitive Affections, Voice Alterations, and Musculoskeletal Affections) and in its manifestations of health gain and well-being (Self-Efficacy and Satisfaction).

The Exhaustion dimension considers the feeling of physical and emotional exhaustion caused by the teaching activity. The importance of this factor is consistent with its involvement in health. According to its authors, it is the factor with the greatest weight in the variance explained. Firstly, this factor corresponds to the burnout dimension of burnout, which is considered to be the central dimension of this syndrome [18,19,20]. It has been pointed out that burnout leads to a deterioration in the functioning of most physiological and psychological systems, thus posing an increased risk of developing respiratory disorders, gastrointestinal disorders, and viral diseases [21,22,23,24,25,26]. It has also been shown to play a mediating role between the experience of stress and the development of depressive disorders [20,27]. Some authors point out that burnout is the decisive element in the negative spirals, or loss of health, as defined in the Job Demands and Resources Model (JDR), indicating that the demands placed on the professional are excessive and that more organisational, material and training resources are required to reduce the teacher’s load [28,29,30]. In this sense, burnout is a consequence of experiencing a situation of overload and is therefore considered an indicator of harmful working conditions [20,31,32].

The Satisfaction dimension refers to satisfaction with the teaching profession, the enjoyment and energy to carry it out, and the feeling of happiness in being a teacher. It is a factor that captures the affective well-being related to work. It is a factor close to engagement, with dimensions of vigour, enjoyment, and absorption, and also to flow, as it assesses a positive affective and motivational relationship with work. In studies on burnout, satisfaction is a factor that correlates negatively with burnout and depersonalisation [18]; it has also been shown to decrease the chances of generating stress [33] and to increase empathy towards and satisfaction of the recipients [34]. Satisfaction in teaching is related to emotions of joy, pride, and enjoyment, i.e., the presence of positive emotions. It is, therefore, a factor that indicates well-being and an optimal state for the development of new competencies and nurturing interpersonal relationships [35,36].

The Voice Alterations dimension considers the presence of physical sensations of discomfort related to the voice, specifically: aphonia, or loss of voice, vocal fatigue, and discomfort in the neck, these aspects being frequent in the teaching profession [37,38,39].

The dimension Musculoskeletal Affections refer to muscular discomfort mainly with the spine and back. This musculature is the most affected, with the most frequent damage being: contractures, cervical or back pain, herniated discs, and lumbago [40,41,42]. It is related to associated with sustained stressful situations based on stress and somatisation processes [37,43].

The Cognitive Affections dimension assesses the presence of dysfunctions in the cognitive abilities of concentration, memory, distractions, and obsessive thinking. This symptomatology is a consequence of the high concentration of glucocorticoids in the hippocampus caused by the experience of emotional exhaustion and distress [44,45]. On the other hand, it is one of the negative effects that participate in the generation of negative spirals of health loss. The professional perceives that their cognitive capacities, essential for the regulation and management of teaching, have diminished, i.e., their professional competence, and this, in turn, produces an increase in the feeling of vulnerability and insecurity, which again increases the experience of distress and the consequent affectation of cognitive capacities. This spiralling process can lead to a crisis of professional efficacy or to the development of Job Burnout Syndrome (WBS) [21,29,46].

The Self-efficacy dimension assesses the teacher’s perception of his or her ability to achieve positive and meaningful results, as well as his or her assessment of his or her professional competence and capabilities. It indicates a positive affective state in relation to teaching, which facilitates the generation of gain spirals [47]. It is considered a central element in identifying gain or loss spiral processes. Numerous studies indicate its relationship with the physical and psychological well-being of teachers and with a good quality of interpersonal relationships. Furthermore, various studies consider it to be an important preventive element in professional health [48,49,50]. A broad conception of the term self-efficacy, as confidence in one’s own competencies, is chosen, since the more restrictive conception of the term self-efficacy requires reference to overcoming obstacles [51]. 

Several models attempt to determine which variables decrease the likelihood that individuals will experience symptoms that deteriorate their occupational health. These include Kobasa’s model [52,53], which indicates that individuals with high scores on resilient personality dimensions have better characteristics in the face of stress at work or in everyday life, and the Coherence Construct developed by Antonovsky [54], which focuses on exploring the origin of health rather than explaining the causes of illness.

In this study, the construct of Effective Personality [55] was used, as it is a model that integrates personal and socio-affective competencies, establishes a related structure between them, and factors them into four broad dimensions that are interrelated to each other. In the construct, these variables are grouped around four categories or dimensions: Self-esteem, Labor Self-Realisation, Resolute Self-efficacy, and Social Self-Realisation.

The Self-esteem dimension measures indicators of self-concept and self-worth and integrates valuational aspects of the person such as good knowledge and appreciation of oneself, a high valuation and confidence in one’s cognitive–emotional and social resources, and accurate recognition of one’s limitations. The Labor Self-Realisation dimension highlights the relationship between personal knowledge and the attribution of success to a defined and positive capacity and effort, intrinsic motivation, and achievement in the activities of daily life. The Resolving Self-efficacy dimension refers to the ability to cope with problems and challenges. It refers to decision-making and coping with problems. Finally, the Social Self-Realisation dimension involves communication skills, assertiveness, and empathy. This dimension highlights the link between self-perceived ability or competence to establish and maintain relationships with others and expectations of success in future social relationships.

Although the variables of the Efficacy Personality model have been the object of specific research relating them to aspects linked to health [14,49,56,57], in this study, these variables are considered to form a unitary construct. 

To these advantages of the model must be added its application in research that has allowed the construction of assessment instruments in different contexts, the establishment of multivariate modal typologies in different ages, as well as the development of intervention programmes developed to work jointly with each of the dimensions assessed.

We aimed to analyse the causal relationship between the dimensions of the Efficacy Personality construct and the dimensions that make up the model of Teachers’ Occupational Health. The aim was to verify that the dimensions of the effective personality construct are mainly related to the dimensions of gain and loss of health in the teaching work environment.

Specifically, each of the dimensions of Efficacy Personality (Self-esteem, Labor Self-Realisation, Resolute Self-Efficacy and Social Self-Realisation) were analysed with each of the dimensions of Teachers’ Occupational Health (Exhaustion, Satisfaction, Voice Disturbance, Musculoskeletal Disorders, Cognitive affectations, and Self-efficacy).

It was hypothesised that there is a positive influence between the Efficacy Personality and the manifestations of health gain (Self-efficacy and Satisfaction) and a negative influence with those of loss (Exhaustion, Cognitive Affections, Musculoskeletal Disorders, and Voice Disturbance).

## 2. Method

This research was based on a descriptive, correlational, and inferential cross-sectional design [58]. In order to obtain information about the dimensions present in the constructs of Effective Personality and Teacher Occupational Health, online questionnaires were applied.

With the data obtained, analyses were carried out to verify the relationship of dependence of the dimensions present in both constructs through correlations, regressions, and structural equations.

Informed consent was obtained from the study participants, and the principles of confidentiality, voluntariness, data protection, and ethical standards for this type of study were observed.

### 2.1. Participants

The inclusion criterion for participants was to belong to the population of non-university teachers in the 435 public schools of the Principality of Asturias, made up of 11,796 people (72.5% female and 27.5% male). In this research, an informatic application has been used to ensure that only the target population, teachers from public schools in the Principality of Asturias, who can only fill in the form once, could participate in the study. Another criterion for inclusion was the voluntary nature of participation.

The final sample consisted of 700 teachers, but one teacher was excluded for completing only one of the questionnaires. The 699 participants constituted a representative sample of the study population at a confidence level of 95% and with a margin of error of 5%. Their distribution according to the different socio-demographic variables is shown in Table 1. As can be seen in Table 1, the distribution of the sample is consistent with the population in terms of the variables gender, affiliation, educational stage, and location of the school, which can be considered a guarantee of the representativeness of the sample.

### 2.2. Instruments

Two questionnaires were used for this study:

- Teaching Health Questionnaire (CSD) by Fernández-Puig, Longas, Chamarro and Virgili [17]. It consists of 23 items integrated into six dimensions or factors:

Exhaustion: it has 3 items and a Cronbach’s Alpha reliability of 0.87. It considers the feeling of physical and emotional exhaustion caused by carrying out the teaching activity (for example: item 12. I feel physically exhausted at the end of my workday). 

Satisfaction: consists of 5 items with a Cronbach’s Alpha reliability of 0.79. It includes the effective well-being related to work (for example: item 10. I have a good time at work.).

Voice disturbance: has 3 items and a Cronbach’s Alpha reliability of 0.82. It considers the presence of physical sensations of discomfort related to the voice, specifically: aphonia, or loss of voice; vocal fatigue, and discomfort in the neck (for example: item 8. I feel hoarse or dysphonic).

Musculoskeletal disorders: has 3 items and a Cronbach’s Alpha reliability of 0.73. They refer to muscular discomfort mainly with the spine and back (for example: item 2. My back hurts from the activity I do.).

Cognitive affectations: it has 4 items and a Cronbach’s Alpha reliability of 0.71. It assesses the presence of dysfunctions in the cognitive abilities of concentration, memory, distractions, and obsessive thinking (for example: item 11. There are times when I have more distractions than usual.).

Self-efficacy: consists of 5 items and a Cronbach’s Alpha reliability of 0.71. It is related to the teacher’s perception of his or her ability to obtain positive and significant results, as well as his or her assessment of his or her professional competence and capabilities. (for example: item 22. When I finish a job, I am often happy with the results.).

The response format of this questionnaire is a Likert-type scale with five response options: 1 = Strongly Disagree; 2 = Disagree; 3 = Neither Agree nor Disagree; 4 = Agree; 5 = Strongly Agree. It can be applied individually or collectively and lasts about 10 min. It can be considered as an evaluation tool that includes the most relevant aspects in the evaluation of teachers’ health and that is framed in the current health surveillance policies.

- Questionnaire of Effective Personality-Adults (CPE-A) by Castellanos, Martín-Palacio, and Dapelo [59]. The CPE-A consists of 30 items integrated into four dimensions that define the Efficacy Personality model:

Self-esteem: consists of 8 items and has a Cronbach’s Alpha reliability of 0.71. It assesses knowledge and appreciation of one’s physique, confidence in one’s cognitive–emotional and social resources, and a recognition of one’s limitations. (for example: item 3. I feel very good about my physical appearance.).

Laboral Self-Realisation: has 8 items and a Cronbach’s Alpha reliability of 0.78. It analyses personal knowledge and attribution of success to a defined and positive ability and effort, intrinsic motivation, and achievement expectancy. (for example: item 5. I am successful in a task because I work hard to do a good job.).

Resolute Self-Efficacy: presents 5 items with a Cronbach’s Alpha reliability of 0.59. It analyses effective coping with challenges and appropriate decision making. (for example: item 12. To make a decision I gather all the information I can find).

Social Self-Realisation: has 9 items and a Cronbach’s Alpha reliability of 0.83. It assesses the person’s communication, assertiveness, and empathy, as well as their relationships with their environment. (for example: item 6. I make friends easily).

The response format of this questionnaire is a Likert-type scale with five response options corresponding to: 1 = Never; 2 = Few times; 3 = Sometimes; 4 = Many times; 5 = Always. It can be applied individually or collectively and lasts about 15 min. In short, it is an instrument that allows an approximation to the characterisation of the dimensions that make up the Effective Personality construct and makes it possible to identify strengths and personal/group requirements that can guide the intervention in programmes following the needs detected.

### 2.3. Procedure

To carry out this study, a form was created using the Microsoft Forms application in Office 365. This application makes it possible to create questionnaires, surveys, and personalised records and share them with the users of an organisation and export the results to Excel in a completely anonymous and confidential way. In addition, the use of the application ensures that only the subjects to whom the study was addressed, teachers from public schools in the Principality of Asturias, could participate in the study, who could only complete the form once.

This form contained the items of the two reference instruments used in the study: the Efficacy Personality Questionnaire-Adults (CPE-A) and the Teacher Health Questionnaire (CSD).

Through the area of Occupational Health, within the Administrative Management and Labour Relations Service of the Regional Ministry of Education and Culture, the anonymous and voluntary collaboration of the teaching staff was requested through the Educastur e-mail, emphasising the scientific purpose of the study and the confidential and anonymous nature of the responses.

The response time was estimated at less than 30 min and data collection was carried out over a period of two school months.

This study complies with the ethical criteria established in the Code of Conduct of the Ethics and Deontology Committee of the Complutense University, approved by the Governing Council on 11 June 2008, and the research procedure followed does not contradict any of these criteria.

### 2.4. Analysis of Data

The SPSS 25.0 and AMOS 24.0 programs were used for the statistical treatment of the data. The independent variable analysed was Efficacy Personality, and the dependent variable was Occupational Health. In order to meet the research objective, a descriptive analysis of both questionnaires was carried out to find out the levels of Efficacy Personality and Occupational Health. To determine the relationship between both models, bivariate Pearson correlations were performed between global and specific scores, as well as multiple linear regressions (by successive steps) to determine the percentage explained by the factors of Efficient Personality in each of the factors of Occupational Health, taking into account the Bonferroni correction and structural equation analysis to determine the influence of Efficacy Personality on the Occupational Health model. The structural equation modelling (SEM) analysis was carried out using the *Maximum Likelihood* procedure (previously checking multivariate normality). In addition, a bootstrap of 10,000 samples was used. The fit indicators used were the following

- CMIN/DF indicates the absolute fit of the model and values below 3 are considered acceptable [60].
- GFI Goodness of Fit Index ranges from 0 to 1 and considers models above 0.90 as adequate [61].
- CFI Comparative Fit Index is one of the most widely used and best performing relative fit indices [62], it also ranges from 0 to 1, with a value of 0.90 being the minimum required to defend the model [63].
- NFI Normed Fit Index assesses the decrease in the χ^2^ statistic of the adopted model with respect to the base model. It must reach a minimum value of 0.90
- SRMR Standardised Root Mean-Square is a measure of the amount of model error, indicating a good fit with values below 0.05 [64].

## 3. Results

Table 2 shows the descriptive analyses of the participant’s scores on the two instruments. It can be seen that the mean scores obtained in Occupational Health and Efficacy Personality are above the theoretical mean in all cases, except for Voice Alterations, where they are below. Furthermore, it is observed that both skewness and kurtosis are between ±2 values, which indicates normality of the variables according to authors such as Pérez [65].

In order to determine the relationship between the constructs of Effective Personality and Teacher Occupational Health a Pearson correlation analysis was carried out when it was found that the variables were normally distributed, as indicated by the skewness and kurtosis. The results of which are shown in Table 3, where it can be seen that the correlations found between the total scores of the Effective Personality construct and the Teacher Occupational Health construct are positive and significant (*p* < 0.001).

When analysing the relationships between the variables that make up both constructs, positive relationships were found, being equally as significant (*p* < 0.001) between the Satisfaction and Self-efficacy dimensions with all the dimensions of the Efficacy Personality. In contrast, negative relationships were found between the dimensions of Burnout and Cognitive Disturbances with all the factors of Effective Personality (moderately). Additionally, to a lesser extent, between the dimensions Musculoskeletal Affections and Voice Alterations with Self-esteem, and Voice Alterations is also negatively related to Resolute Self-efficacy.

Secondly, the results obtained in the multiple linear regression analyses to determine which dimensions of Efficacy Personality influence the dimensions of Occupational Health are presented.

First, regression was performed on the satisfaction factor. It was found that, on the whole, the assumptions for the application of multiple linear regression are met, with the exception of normality, so the results were be taken with caution. There were significant correlations between all variables *p* < 0.05 indicating an adequate linear association. The Durbin-Watson value = 1.80, a value between 1.5 and 2.5, so independence is met. The eigenvalues of the variance inflation factor (VIF) are between 0.01 and 3.97, all less than 10, which ensures non-multicollinearity. The standardised residuals conform to a homoscedastic distribution. The Kolmogorv-Smirnov (*K-S*) test showed that the normality assumption is not met (*p* < 0.05). The regression showed that in the Satisfaction dimension, the dimensions of Labor Self-Realisation, Social Self-Realisation, and Resolute Efficacy were significant predictors, explaining 24.3% of the variance, in all cases with a positive relationship (Table 4).

Secondly, the regression was carried out on the Self-efficacy factor. After verifying that, on the whole, the assumptions for the application of multiple linear regression were met, except for normality, the results were taken with caution. There are significant correlations between all variables *p* < 0.05, indicating an adequate linear association. The Durbin-Watson value = 1.92, a value between 1.5 and 2.5, so independence is met. The eigenvalues of VIF are between 0.01 and 3.97, all less than 10, which ensures non-multicollinearity. The standardised residuals conform to a homoscedastic distribution. The *K-S* test shows that the normality assumption is not met (*p* < 0.05). The regression showed that in Self-efficacy, the dimensions of Labor Self-Realisation, Self-esteem, and Social Self-Realisation were significant predictors explaining 40.2% of the variance, in all cases with a positive relationship (Table 5).

Thirdly, regression was performed on the Exhaustion factor. After verifying that, on the whole, the assumptions for the application of multiple linear regression were met, except for normality, the results were taken with caution. There are significant correlations between all variables *p* < 0.05 indicating an adequate linear association. The Durbin-Watson value = 1.96, a value between 1.5 and 2.5, so independence is met. The eigenvalues of VIF are between 0.01 and 3.98, all less than 10, which ensures non-multicollinearity. The standardised residuals conform to a homoscedastic distribution. The *K-S* test shows that the normality assumption is not met (*p* < 0.05). The regression showed that with respect to Exhaustion, the dimensions of Self-esteem, Labor Self-Realisation, and Resolute Self-Efficacy were significant predictors explaining 7.5% of the variance, in a negative sense in all of them except for Labor Self-Realisation (Table 6).

Fourthly, the regression was carried out on the factor Voice Alterations. After verifying that there were no significant correlations between this factor and Labor Self-Realisation and Social Self-Realisation, these were excluded from the analysis. The rest of the factors, as a whole, meet the assumptions for the application of linear regression, with the exception of normality, so the results will be taken with caution. There are significant correlations between all variables at *p* < 0.05 indicating an adequate linear association. The Durbin-Watson value = 1.91, a value between 1.5 and 2.5, so independence is met. The eigenvalues of VIF are between 0.01 and 1.99, all less than 10, which ensures non-multicollinearity. The standardised residuals conform to a homoscedastic distribution. The *K-S* test shows that the normality assumption is not met (*p* < 0.05). The regression showed that only the dimension Self-esteem was a significant predictor of voice disturbances, explaining 1.2% of the variance, being negative (Table 7).

Fifthly, the Musculoskeletal Affections factor was regressed. After verifying that there were no significant correlations between this factor and Labor Self-Realisation and Social Self-Realisation, these were excluded from the analysis. The rest of the factors, as a whole, meet the assumptions for the application of linear regression, with the exception of normality, so the results were taken with caution. There are significant correlations between all variables *p* < 0.05 indicating an adequate linear association. The Durbin-Watson value = 1.93, a value between 1.5 and 2.5, so independence is met. The eigenvalues of VIF are between 0.01 and 1.99, all less than 10, which ensures non-multicollinearity. The standardised residuals conform to a homoscedastic distribution. The *K-S* test shows that the normality assumption is not met (*p* < 0.05). The regression showed that in Musculoskeletal Affections, only the dimension Self-esteem was a significant predictor, explaining 1.3% of the variance, in a negative direction (Table 8).

Finally, in sixth place, the regression on the Cognitive Affections factor was carried out. After verifying that, on the whole, the assumptions for the application of multiple linear regression were fulfilled, except for normality, the results were taken with caution. There are significant correlations between all variables *p* < 0.05 indicating an adequate linear association. The Durbin-Watson value = 2.08, a value between 1.5 and 2.5, so independence is met. The eigenvalues of VIF are between 0.01 and 4.96, all less than 10, which ensures non-multicollinearity. The standardised residuals conform to a homoscedastic distribution. The *K-S* test shows that the normality assumption is not met (*p* < 0.05). The regression showed that in relation, to the Cognitive Affections, the dimensions Self-esteem, Resolute Self-Efficacy, Social Self-Realisation, and Labor Self-Realisation were significant predictors, explaining 11.9% of the variance, in a negative sense for all of them except for Labor Self-Realisation (Table 9).

**Figure 1:**
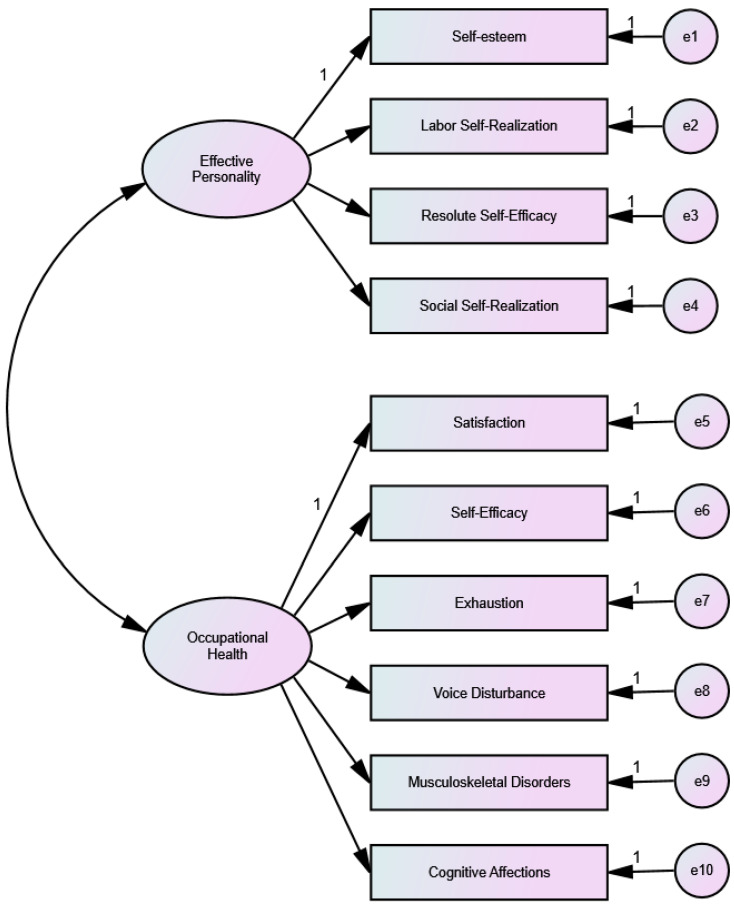
Model 1: Effective Personality Relationship with Occupational Health.

**Figure 2:**
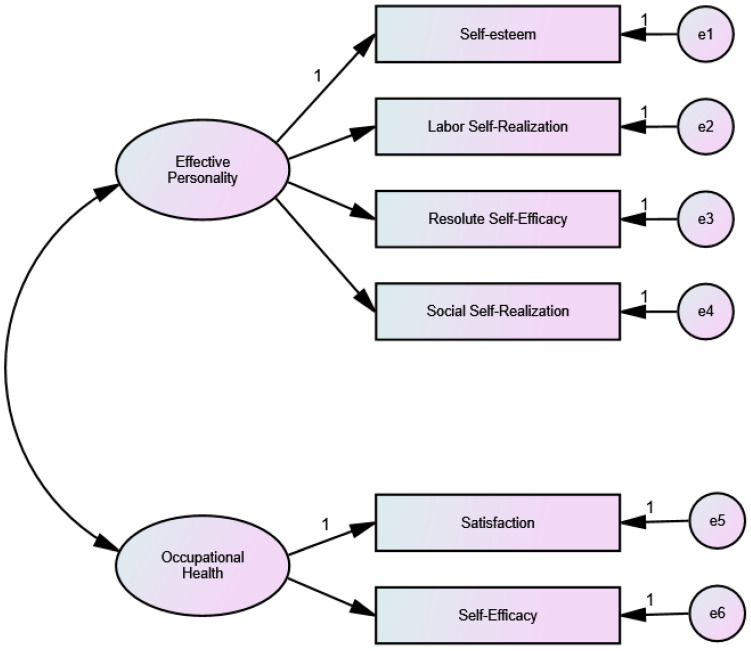
Model 2: Effective Personality Relationship with Satisfaction and Self-efficacy factors.

In order to test the possible relationships between the dimensions of Efficacy Personality and Occupational Health, a Structural Equation model was used. As an initial step in proposing the model, a Confirmatory Factor Analysis was performed to obtain appropriate fit indices. Model 1, relating the four Efficacy Personality factors to the six Occupational Health factors (Figure 1), and Model 2 relating Efficacy Personality to the Occupational Health Satisfaction and Self-Efficacy factors (Figure 2), more related to health gain aspects, were tested. These analyses were performed using the Maximum Likelihood procedure after checking that the multivariate normality assumption indicated a Mardia coefficient of less than 70 (Model 1 r.c. = 12.94; Model 2 and SEM r.c. = 17.93). Authors such as Rodríguez and Ruiz [66] consider that this is the method that provides the best results even if there is a distance from the normality assumption.

When testing both models (Table 10) it was found, on the one hand, that in Model 1, the Efficacy Personality questionnaire showed adequate composite reliability (CR) and average variance extracted (AVE), while Occupational Health showed inadequate CR and AVE. In addition, the fit indices show a poor fit of the data with this model. On the other hand, in Model 2, both the Efficacy Personality and the Occupational Health questionnaires have adequate HR and AVE, and the indices also show a good fit to the model, with the exception of the CMIN/DF index, possibly due to the sample size.

**Figure 3:**
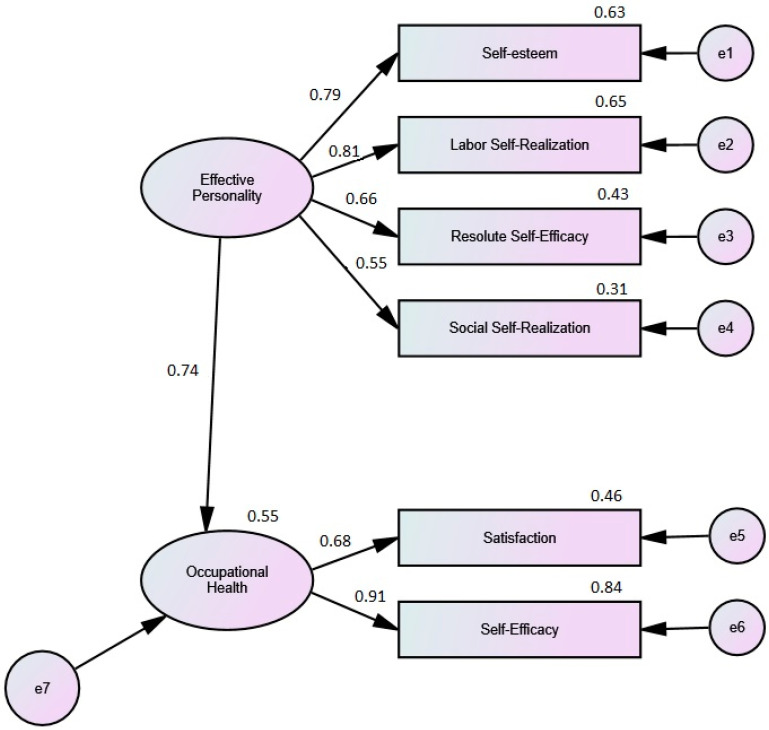
Standardised Regression Weights of the SEM: Model of Structural Equations of Effective Personality Relationship with Satisfaction and Self-efficacy Factors.

When analysing the structural equation model (SEM) of the relationship between Efficacy Personality and Occupational Health (Figure 3), it was observed that there is a significant, direct, and positive relationship between both constructs (standardised regression weight = 0.74; *p* < 0.001). Furthermore, the *R*^2^ indicates that the model explains 55.0% of the variance in Occupational Health in the aspects related to health gain (Satisfaction and Self-efficacy). This indicates the clear protective role of the Efficacy Personality on Occupational Health.

## 4. Discussion

This paper aimed to analyse the relationship between Efficacy Personality and Teacher Occupational Health and, more specifically, between the dimensions that make up the first construct and the dimensions of Satisfaction, Self-efficacy, and burnout of the second.

As a starting hypothesis, it was proposed that there is a positive influence between the Efficacy Personality and the manifestations of health gain (Self-efficacy and Satisfaction) and a negative influence with the manifestations of health loss (Exhaustion, Cognitive Affections, Musculoskeletal Affections, and Voice Alterations).

In the light of the results obtained, it can be indicated that this hypothesis is partially confirmed, since there is a clear relationship between the dimensions of Efficacy Personality and the health gain factors, and to a lesser extent with the loss factors (these results are not statistically significant).

More specifically, positive correlations were found between manifestations of health gain and all dimensions of the Efficacy Personality. This indicates that high Self-Efficacy and Satisfaction correspond to high levels of Self-Esteem, Labor Self-Realisation, Resolute Efficacy, and Social Self-Realisation. Therefore, teachers who perceive themselves as having the good professional ability and feel satisfied with their teaching work are people with high self-esteem, with the ability to make adequate internal attributions of professional achievements, have high expectations, are effective in problem-solving, and have adequate social skills that provide them with a consolidated social support network.

This positive relationship is corroborated by linear regression analyses that show the influence of the Efficacy Personality dimensions on Occupational Health. In addition, structural equation analysis confirms the influence of Efficacy Personality on the factors of Self-efficacy and Satisfaction.

The above results are in line with Kobasa [53], who found that subjects with high scores on resilient personality factors show higher levels of protection against work stress. This model characterises a resilient personality by three basic components: engagement (the ability to be involved in life activities), control (the person’s ability to take control of his or her life), and challenge (the ability to see change as a challenge rather than a threat). It is worth noting that a wider range of dimensions are integrated into the Efficacy Personality construct.

More recent studies also confirm the influence of personal resources on teacher satisfaction and well-being [7,67,68,69,70,71,72,73]. Other authors also point to the importance of these resources for teacher performance [74].

We also found agreement with other research [75] that confirms the role of self-esteem in promoting behaviours related to occupational health. This was also observed with those that relate self-efficacy with teacher motivation [76,77,78,79], as well as studies that confirm that this relationship is positive, finding that teachers with high levels of self-efficacy are more motivated and more enthusiastic about the teaching process [80].

Other authors also explain the importance of the positive relationships found [81], indicating that commitment to the student, having good instructional strategies, and effective classroom management have a positive impact on teachers’ happiness.

As for the manifestations of loss of health, it has been found that it correlates negatively, although low and not significantly, with Effective Personality. The most relevant relationships were found between Cognitive Affections and burnout with the Effective Personality scale.

By deduction of the profiles found in the teachers who presented high values in the dimensions of Efficacy Personality, we can point out the following notes. Specifically, about the factor Cognitive Affections, it has been found that the subjects with the greatest personal resources, those who obtain the highest scores in the dimensions of the Efficacy Personality construct, have the lowest rates of cognitive affect. It should be noted that this dimension is one of the main factors involved in the generation of negative spirals of health loss in the teaching profession (crisis of professional efficacy and development of burnout).

On the other hand, the Exhaustion factor shows that those teachers who present a greater presence of personal resources (Self-concept, Self-esteem, Resolution self-efficacy, and Social Self-Realisation) obtained lower scores in the Exhaustion factor of the teacher occupational health model, giving, therefore, these resources an important moderating and protective role. It should be noted in this respect that the burnout factor is a decisive element in the spirals of health loss in the teacher occupational health model (illness processes and depressive disorders) and that it also corresponds to the central dimension of the burnout syndrome.

This indicates that teachers’ perception of a decrease in their cognitive abilities (concentration problems, lack of memory, frequent distractions, and obsessive thinking) is negatively related to their self-esteem, self-efficacy in solving problems, and Social and Labor Self-Realisation, causing a significant feeling of vulnerability and insecurity. In addition, a high state of burnout leads to a low sense of self-esteem, low motivation, a low tendency to make positive causal attributions, and developing low expectations of success.

These results are in line with those found by other authors, who state that people with low levels of Self-esteem feel less effective in factors such as communication, understanding, attention, and excellence [82,83]. In addition, low levels of burnout are found, firstly, in teachers who have a high profile of efficacy, attributions, and positive expectations of success [84]. Secondly, these levels are also found in teachers with high sociability, well-being, and self-control [7]. This shows the importance of the factors of the Efficacy Personality construct since several of them are part of its dimensions (Self-esteem, Attributions, Expectations, and Sociability).

## 5. Conclusions

The results confirm the relationship between the dimensions of the Efficacy Personality construct and the dimensions that make up the gain in Teacher Occupational Health (Self-Efficacy and Satisfaction). No such clear evidence was found to the dimensions of loss (Exhaustion, Cognitive Affections, Musculoskeletal Affections, and Voice Alterations).

Therefore, we can consider the protective function of the Efficacy Personality on Teachers’ Occupational Health.

It is evident that the strengthening of personal competencies and resources is a first-order necessity for an effective and healthy exercise of the teaching profession. It is essential to promote the development of personal health resources in the teaching profession, both at the level of initial and in-service teacher training.

In this sense, the construct of Effective Personality has developed programmess that allow a continuous education and training of the different dimensions that constitute it and that have already been implemented in different educational and professional contexts [85].

Education and training should be complementary to the analysis of other organisational and group aspects of the work context since together they determine an efficient, effective, and healthy professional performance.

## 6. Limitations and Future Directions

Below, we point out the main limitations that we consider to be present in this study and that should be taken into account when interpreting and generalising the results.

The first limitation refers to the type of cross-sectional design employed. It would be interesting to develop a longitudinal design that would allow us to study the evolution of the variables analysed, and thus be able to establish causal relationships.

Secondly, another limitation is the lower value of the reliability coefficients of the “Resolute self-efficacy” factor, possibly due to the smaller number of items that make up this dimension. Despite this, it is within acceptable values for the statistical treatment of the data. As a line for the future, it would be advisable to increase the number of items and reliability of this factor.

Concerning the sample, it would be desirable to have a larger number of participants as well as to extend it to the different regions of the country.

Another limitation is the fact that the assumption of normality in the multiple linear regression test was not met, which means that the results should be interpreted with caution. However, the other assumptions were met.

On the other hand, no clear influence has been found for health loss factors, so it would be necessary to look into this aspect in greater depth.

Given the importance of the results, it would be advisable for future studies to extend the sample to other cultural contexts and other levels of teaching staff, such as university teachers, and other variables such as gender and age.

It would also be interesting to know to what extent these dimensions are present in each of the multivariate typologies of the Efficacy Personality construct. This study will allow us to deepen our knowledge of needs and opportunities for improvement with resources that must necessarily form part of teaching competencies.

In the future, it would also be interesting to apply effective personality development programs that train the different dimensions and to monitor their effectiveness with this population [85].

## Figures and Tables

**Table 1:** Descriptive statistics for the population and the sample.

Variable	Population	Sample
N	11.796	699
Age		*M* = 47.65; *SD* = 8.68
Sex		
Female	8.562 (72.5%)	506 (72.6%)
Male	3.234 (27.5%)	193 (27.6%)
Affiliation		
Career or permanent civil servants	4198 (64.4%)	497 (71.1%)
Interim or permanent civil servants	7598 (35.6%)	202 (28.9%)
Educational stage		
Teachers	5615 (47.6%)	275 (39.3%)
Secondary	4976 (42.2%)	297 (42.5%)
Vocational Education	730 (6.2%)	89 (12.7%)
Other education	475 (4.0%)	38 (5.5%)
Location of the centre		
Large population centres (more than 100,000 inhabitants)	129 centres (29.65%)	289 (41.5%)
Small population centres (between 10,000 and 100,000 inhabitants)	194 centres (44.60%)	277 (39.5%)
Rural area (in population centres with less than 10,000 inhabitants)	112 centres (25.75%)	133 (19.0%)

**Table 2:** Descriptive of Occupational Health and Efficacy Personality.

	N Items	Theoretical *Mean*	Empirical *Mean*	*SD*	*Minimun*	*Maximum*	*Skewness*	*Kurtosis*
CDS satisfaction	5	15	19.63	3.84	5	25	−0.73	0.39
CDS self-efficacy	5	15	20.28	2.78	9	25	−0.74	1.19
CDS exhaustion	3	9	9.86	3.07	3	15	−0.24	−0.62
CDS voice alterations	3	9	7.95	2.95	3	15	0.11	−0.77
CDS musculoskeletal affections	3	9	9.51	3.28	3	15	−0.22	−0.82
CDS cognitive affections	4	12	13.32	3.28	4	20	−0.40	−0.14
CDS total	23	69	77.27	12.31	38	115	0.02	0.23
CPE-A: Self-esteem	8	24	29.77	4.01	16	40	−0.28	0.58
CPE-A: Labour Self-Realisation	8	24	33.15	3.64	21	40	−0.17	0.02
CPE-A: Resolute Self-Efficacy	5	15	19.73	2.70	8	25	−0.40	0.51
CPE-A: Social Self-Realisation	9	27	34.55	5.11	17	45	−0.55	0.33
CPE-A: Total Effective Personality	30	90	117.20	12.15	766	150	−0.19	0.35

**Table 3:** Correlations between Effective Personality and Teacher Health.

	CPE-A: Total Effective Personality	CPE-A: Self-Esteem	CPE-A: Labour Self-Realisation	CPE-A: Resolute Self-Efficacy	CPE-A: Social Self-Realisation
CDS Total	0.45 **	0.41 **	0.32 **	0.36 **	0.32 **
CDS Satisfaction	0.47 **	0.33 **	0.43 **	0.35 **	0.37 **
CDS Self-efficacy	0.59 **	0.50 **	0.60 **	0.39 **	0.39 **
CDS Exhaustion	−0.20 **	−0.24 **	−0.08 *	−0.19 **	−0.14 **
CDS Voice Alterations	−0.08 *	−0.11 **	−0.01	−0.09 *	−0.05
CDS Musculoskeletal Affections	−0.06	−0.11 **	0.03	−0.07	−0.04
CDS Cognitive Affections	−0.30 **	−0.28 **	−0.15 **	−0.28 **	−0.23 **

* *p* < 0.05; ** *p* < 0.01.

**Table 4:** Regression Analysis of the Influence of Effective Personality Factors on Satisfaction.

DV	IV	B	*β*	95% Confidence Interval		*R*	*R* ^2^	*R*^2^ Corrected	Change at *R*^2^
				Lower Limit	Upper Limit				
Satisfaction	(Constant)	0.76		−1.75	3.26	0.49	0.24	0.24	0.01 *
	Labor Self-Realisation	0.29	0.27 *	0.21	0.37				
	Social Self-Realisation	0.16	0.21 *	0.10	0.21				
	Resolute Self-Efficacy	0.19	0.14 *	0.09	0.30				

* Bonferroni correction (*p*-value 0.05/3 tests = 0.016).

**Table 5:** Regression Analysis of the Influence of Effective Personality Factors on Self-Efficacy.

DV	IV	B	*β*	95% Confidence Interval		*R*	*R* ^2^	*R*^2^ Corrected	Change at *R*^2^
				Lower Limit	Upper Limit				
Self-Efficacy	(Constant)	3.16		1.59	4.74	0.63	0.40	0.40	0.01 *
	Labor Self-Realisation	0.35	0.46 *	0.29	0.41				
	Self-esteem	0.11	0.16 *	0.05	0.16				
	Social Self-Realisation	0.07	0.13 *	0.03	0.10				

* Bonferroni correction (*p*-value 0.05/3 tests = 0.016).

**Table 6:** Regression Analysis of the Influence of Effective Personality Factors on Exhaustion.

DV	IV	B	*β*	95% Confidence Interval		*R*	*R* ^2^	*R*^2^ Corrected	Change at *R*^2^
				Lower Limit	Upper Limit				
Exhaustion	(Constant)	14.38		12.24	16.52	0.27	0.07	0.07	0.01 *
	Self-esteem	−0.20	−0.26 *	−0.28	−0.13				
	Labor Self-Realisation	0.13	0.15 *	0.05	0.21				
	Resolute Self-Efficacy	−0.13	−0.12 *	−0.24	−0.03				

* Bonferroni correction (*p*-value 0.05/3 tests = 0.016).

**Table 7:** Regression Analysis of the Influence of Effective Personality Factors on Voice Disturbance.

DV	IV	B	*β*	95% Confidence Interval		*R*	*R* ^2^	*R*^2^ Corrected	Change at *R*^2^
				Lower Limit	Upper Limit				
Voice Alterations	(Constant)	10.37		8.74	12.01	0.11	0.01	0.01	0.01 *
	Self-esteem	−0.08	−0.11 *	−0.14	−0.03				

* *p* < 0.05.

**Table 8:** Regression Analysis of the Influence of Effective Personality Factors on Musculoskeletal Disorders.

DV	IV	B	*β*	95% Confidence Interval		*R*	*R* ^2^	*R*^2^ Corrected	Change at *R*^2^
				Lower Limit	Upper Limit				
Musculoskeletal Affections	(Constant)	12.32		10.51	14.14	0.11	0.01	0.01	0.01 *
	Self-esteem	−0.09	−0.11 *	−0.15	−0.03				

* *p* < 0.05.

**Table 9:** Regression Analysis of the Influence of Effective Personality Factors on Cognitive Affections.

DV	IV	B	*β*	95% Confidence Interval		*R*	*R* ^2^	*R*^2^ Corrected	Change at *R*^2^
				Lower Limit	Upper Limit				
Cognitive Affections	(Constant)	21.75		19.44	24.07	0.34	0.12	0.11	0.01 *
	Self-esteem	−0.16	−0.19 *	−0.24	−0.07				
	Resolute Self-Efficacy	−0.23	−0.19 *	−0.34	−0.13				
	Social Self-Realisation	−0.08	−0.12 *	−0.13	−0.03				
	Labor Self-Realisation	0.11	0.12 *	0.02	0.19				

* Bonferroni correction (*p*-value 0.05/4 tests = 0.0125).

**Table 10:** Model fit and validity and reliability indices.

	**Model 1**		**Model 2**	
CMIN/DF	19.67		8.96	
GFI	0.80		0.97	
CFI	0.73		0.96	
NFI	0.72		0.95	
SRMR	0.12		0.04	
	**Effective Personality**	**Occupational Health**	**Effective Personality**	**Occupational Health**
CR	0.80	0.02	0.80	0.78
AVE	0.51	0.26	0.50	0.65
MSV	0.58	0.58	0.55	0.55
MaxR(H)	0.82	0.78	0.83	0.86
Effective Personality	0.71	0.76 ***	0.71	0.74 ***
Occupational Health		0.51		0.81

*** Correlation *p* < 0.001.

## Data Availability

The datasets used and/or analysed during the current study are available from the corresponding author.
